# Exploring Differentially Expressed Genes and Natural Antisense Transcripts in Sheep (*Ovis aries*) Skin with Different Wool Fiber Diameters by Digital Gene Expression Profiling

**DOI:** 10.1371/journal.pone.0129249

**Published:** 2015-06-15

**Authors:** Yaojing Yue, Tingting Guo, Jianbin Liu, Jian Guo, Chao Yuan, Ruilin Feng, Chune Niu, Xiaoping Sun, Bohui Yang

**Affiliations:** Lanzhou Institute of Husbandry and Pharmaceutical Sciences, Chinese Academy of Agricultural Sciences, Jiangouyan Street, Lanzhou, China; University of North Carolina at Charlotte, UNITED STATES

## Abstract

Wool fiber diameter (WFD) is the most important economic trait of wool. However, the genes specifically controlling WFD remain elusive. In this study, the expression profiles of skin from two groups of Gansu Alpine merino sheep with different WFD (a super-fine wool group [FD = 18.0 ± 0.5 μm, n= 3] and a fine wool group [FD=23.0±0.5μm, n=3]) were analyzed using next-generation sequencing–based digital gene expression profiling. A total of 40 significant differentially expressed genes (DEGs) were detected, including 9 up-regulated genes and 31 down-regulated genes. Further expression profile analysis of natural antisense transcripts (NATs) showed that more than 30% of the genes presented in sheep skin expression profiles had NATs. A total of 7 NATs with significant differential expression were detected, and all were down-regulated. Among of 40 DEGs, 3 DEGs (AQP8, Bos d2, and SPRR) had significant NATs which were all significantly down-regulated in the super-fine wool group. In total of DEGs and NATs were summarized as 3 main GO categories and 38 subcategories. Among the molecular functions, cellular components and biological processes categories, binding, cell part and metabolic process were the most dominant subcategories, respectively. However, no significant enrichment of GO terms was found (corrected P-value >0.05). The pathways that were significantly enriched with significant DEGs and NATs were mainly the lipoic acid metabolism, bile secretion, salivary secretion and ribosome and phenylalanine metabolism pathways (P < 0.05). The results indicated that expression of NATs and gene transcripts were correlated, suggesting a role in gene regulation. The discovery of these DEGs and NATs could facilitate enhanced selection for super-fine wool sheep through gene-assisted selection or targeted gene manipulation in the future.

## Introduction

Fine wool sheep, also called Merino, is a world famous sheep breed that is known to produce high-quality fine wool. Fine wool sheep are distributed primarily in Australia, China, New Zealand, South Africa, Uruguay, Argentina and other countries[1]. Wool fiber diameter(WFD) is the most important economic trait of merino sheep and determines 75% of the value of wool fibers. The WFD variation-induced profit accounted for 61% of the total profits of wool [2, 3]. Wool is formed from keratinocytes derived from a progenitor population at the base of the hair follicle(HF)[4]. The morphogenesis and growth of HF in sheep has been extensively studied since the 1950’s and the developmental processes at the cellular level are reasonably well understood [5–7]. Dermal papilla (DP) cells are a population of mesenchymal cells at the base of the HF[8], and provide signals that contribute to specifying the size, shape and pigmentation of the wool[9]. It is well known that WFD is significantly associated with size of DP and matrix in mammals [4, 6, 10–17], and is largely specified post-initiation, during the period of HFs growth and morphogenesis[18]. To illustrate the molecular mechanisms of controlling WFD, the expression profiles of different stage of fetal and adult sheep skin have also been generated by sequencing of expressed sequence tags (ESTs) and cDNA microarray [19, 20]. However, the size of DP and matrix in mammals is also markedly influenced by genetic[10, 11, 21], physiological[13], nutrition[22], hormones[12] during the anagen phase of the hair cycle. Up to now, there are no studies on the molecular mechanisms of controlling WFD during the anagen phase, and the genes specifically controlling WFD remain elusive [23].

Knowledge of the genes controlling development of DP and matrix come from studies of the morphogenesis and cycle of HF of mice and human[24–28], It involves a series of signaling between the matrix and the dermal papill, such as Wnt/beta-catenin, EDA/EDAR/NF-κB, Noggin/Lef-1, Ctgf/Ccn2, Shh, BMP-2/4/7, Dkk1/Dkk4 and EGF[4, 29]. The mutation, epigenetic modification and post-translational modification of any ligand or receptors in these pathways maybe affect WFD [30, 31]. Therefore, in addition to the current efforts on protein-encoding genes, the attention should also be paid to a novel regulatory factor, non-coding RNA (ncRNA), such as micro RNA, long non-coding RNA and natural antisense transcripts (NATs)[32]. Among these ncRNAs, NATs are not only large in quantity but also play important roles in gene expression regulation in organisms [33]. NATs refer to a class of non-coding RNAs that are produced inside organisms under natural conditions and are expressed in many species [34–36]. NATs play an important role in transcript regulation at the mRNA and/or protein levels and in regulating various physiological and pathological processes, such as organ formation, cell differentiation and disease [37, 38]. However, the roles of NATs in controlling WFD have not been described.

The next-generation sequencing (NGS)-based digital gene expression (DGE) profiling technologies developed in recent years constitute a revolutionary change in traditional transcriptome technology. Compared with ESTs and cDNA microarray, the strength of DGE profiling is that it is an "open system" and has better capability to discover and search for new information, providing a new way to identify novel genes and NATs that specifically control WFD [39]. However, the success or effectiveness of the search is largely dependent on the completeness and stitching quality of the genome sequences of the species studied [40]. It is exciting that the International Sheep Genomics Consortium (ISGC) has achieved initial success in assembling the reference sheep genome. The total length of the assembled genome, Sheep Genome v3.1, has reached 2.64 Gb, with only 6.9% gaps [41]. Needless to say, the release of a high-quality sheep genome reference sequence provides important resources for using NGS to study the skin transcriptome of sheep with different WFD and find genes and NATs that specifically control WFD [42, 43].

In this study, NGS-based DGE profiling was conducted to analyze the differentially expressed genes (DEGs) and NATs in the skin of Gansu Alpine fine wool sheep with different WFD. A total of 47 significant DEGs and NATs were detected, including 9 up-regulated genes, 31 down-regulated genes and 7 down-regulated NATs. These DEGs and NATs may be useful in further study on molecular markers of controlling WFD in fine wool sheep.

## Results

### Sequencing and assembly

NGS was performed on 6 individuals of the super fine wool group (sample nos.65505,65530,65540;n = 3) and the fine wool group (sample nos.5Y127,5Y212,5Y339;n = 3), and raw data greater than 5 Mb were obtained for all individuals (Table 1), submitted to the NCBI BioProject database, with the accession number PRJNA274817. There was a 3' adaptor sequence in the raw data, along with small amounts of low-quality sequences and various impurities. Impurity data were removed from the raw data, and clean tags greater than 4.7 Mb were obtained. The distinct tag number of each individual was greater than 0.11 Mb (Table 1).

**Table 1:** Summary of tag numbers based on the DGE data from Gansu Alpine fine wool sheep skin with different WFD.

Sample ID	Raw Data		Clean Tag	
	Total number	Distinct Tag number	Total number	Distinct Tag number
5Y127	5259561	355014	4917435	119886
5Y212	5364832	350472	5033144	117880
5Y339	5145452	336695	4857460	120525
65505	5244848	314710	4930422	110879
65530	5485701	343795	5184375	117642
65540	5282704	373209	4941669	143800

The sheep genome reference sequence database includes 19,346 gene sequences, including 18,940 genes with "CATG" loci and accounting for 97.90% of the total number of genes. The total number of reference tags in the reference tag database is 173,207, including 169,843 unambiguous reference tags, accounting for 98.06% of the total number of tags. All clean tags were compared with reference genes and reference genomes, and the results indicated that 87.45%, 87.32%, 88.63%, 88.97%, 88.72% and 87.38% of the total numbers of clean tags of the 6 individuals from two groups (sample nos. 5Y127, 5Y212, 5Y339, 65505, 65530,and 65540, the same hereinafter), respectively, could be matched to the reference tags; of these, 49.05%, 50.26%, 51.86%, 52.41%, 51.48% and 50.37% of the clean tags could be uniquely located in the reference sequences (sense and antisense), and the ratios of distinct tag were 36.00%, 36.63%, 37.64%, 36.61%, 37.33% and 33.22% (Table 2), respectively; the numbers of unambiguous tag-mapped genes were 10,391, 10,209, 10,666, 10,434, 10,137, 10,298,10763 and 9,965, respectively. (Table 2) These uniquely located sequences indicated that the key genes that regulate the WFD may exist in genes expressed in the individuals with different WFD.

**Table 2:** Summary of unambiguous tag mapping to gene and unambiguous tag-mapped genes (sense & anti-sense).

Sample ID	Unambiguous Tag Mapping to Gene				Unambiguous Tag-mapped Genes	
	Total number	Total % of clean tag	Distinct Tag number	Distinct Tag % of clean tag	number	% of ref genes
5Y127	2412224	49.05%	43158	36.00%	10391	53.71%
5Y212	2529818	50.26%	43175	36.63%	10209	52.77%
5Y339	2519260	51.86%	45369	37.64%	10666	55.13%
65505	2583990	52.41%	40595	36.61%	10434	53.93%
65530	2668978	51.48%	43910	37.33%	10137	52.40%
65540	2489004	50.37%	47775	33.22%	10298	53.23%

There were a large number of possible unknown tags that are in the gene expression profile libraries of these six individuals. Accounting for 12.55% (617,198), 12.68% (638,292), 11.37% (552,279), 11.03% (543,900), 11.28% (585,017) and 12.62% (623,705) of the total number of clean tags were unannotated, respectively; the proportions of distinct tag number of unknown tag were 18.77% (22,503),18.79% (22,152),16.17% (19,493),18.02% (19,975),17.25% (20,292) and 20.43% (29,382), respectively. These results suggested that there were many unknown genes in sheep skin tissue that may also play important roles in the regulation of HF development and wool growth.

### Analysis of the expression profiling of NATs

Sense-antisense regulation is an important method for controlling gene expression. If the clean tags can be matched to the antisense strand of the gene, then it suggests that there are also transcripts for the antisense strand of this gene and that this gene may be subjected to sense-antisense regulation [44]. In the present study, we found that in the gene expression profiling libraries of the 6 individuals of the two groups, the percentages of genes with antisense transcripts were, respectively, 5.83%, 6.01%, 5.91%, 5.84%, 6.00% and 6.20% of the total numbers of the genes in the library, including 247,816 (5.04%), 262,353 (5.21%), 249,595 (5.14%), 247,940 (5.03%), 266,926 (5.15%) and 265,599 (5.37%) tags that could be exactly matched, respectively (Table 3); the number of tag species accounted for, respectively, 13.31%, 13.31%, 13.46%, 13.20%, 13.65% and 11.94% of the total numbers of tag species in the expression profile libraries of the 6 individuals from the 2 groups, including 15,192 (12.67%), 14,966 (12.70%), 15,483 (12.85%), 13,957 (12.59%), 15,289 (13.00%) and 16,346 (11.37%) tag species that could be exactly matched, respectively(Table 3); there were 6472 (33.45%), 6398 (33.07%), 6585 (34.04%), 6227 (32.19%), 6346 (32.80%) and 6758 (34.93%) genes with NATs in the gene expression profiles of the 6 individuals of the 2 groups (Table 3). The sense-antisense transcript ratios of all 6 expression profile libraries exhibited similar trends. The ratios between the tag number, distinct tag number and tag-mapped genes of sense and antisense transcripts were 10:1, 2:1 and 5:3, respectively; the spearman r between the sense and antisense transcripts expression were 0.533 ~ 0.557.

**Table 3:** Summary of unambiguous tag mapping to anti-sense genes and unambiguous tag-mapped anti-sense genes.

Sample ID	Unambiguous tag mapping to anti-sense genes				Unambiguous tag-mapped anti-sense genes	
	Total number	Total % of clean tag	Distinct Tag number	Distinct Tag % of clean tag	number	% of ref genes
5Y127	247816	5.04%	15192	12.67%	6472	33.45%
5Y212	262353	5.21%	14966	12.70%	6398	33.07%
5Y339	249595	5.14%	15483	12.85%	6585	34.04%
65505	247940	5.03%	13957	12.59%	6227	32.19%
65530	266926	5.15%	15289	13.00%	6346	32.80%
65540	265599	5.37%	16346	11.37%	6758	34.93%

### Detection of DEGs, NATs and validation

**Fig 1:**
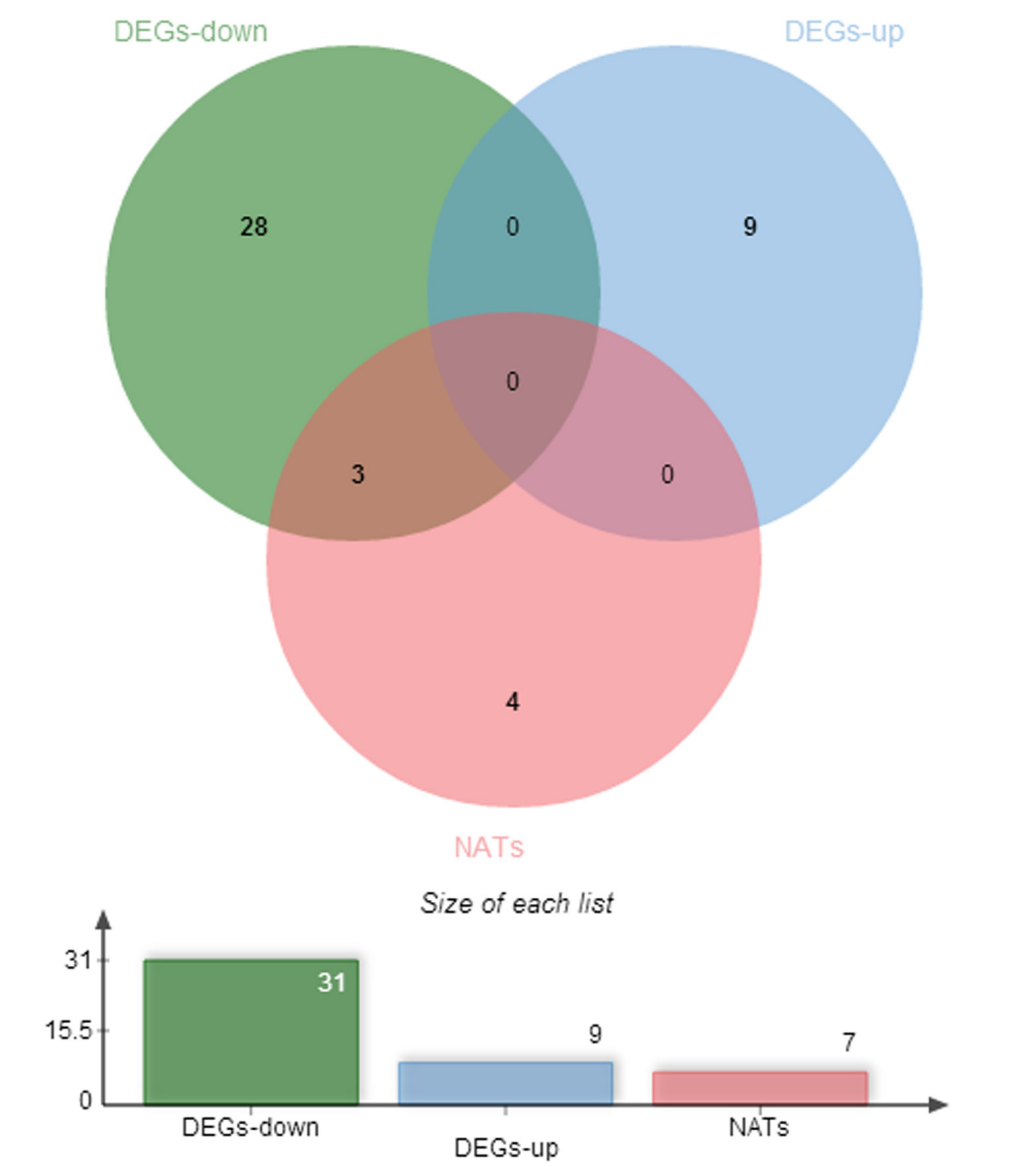
The numbers of DEGs between two groups. Between two groups, there were 9 upregulated genes and 31 downregulated genes and 7 NATs.

The clean tags of all six individuals were aligned with the reference tag database, allowing a maximum of one base mismatch. Unambiguous tags were annotated, and the number of raw clean tags that corresponded to the same gene was counted and then standardized to obtain the standardized expression level of each gene in the skin transcriptome of the 6 individuals. Noiseq software was used to select DEGs, NATs that exhibited different expression levels between S and F group. However, there were only 47 significant DEGs and NATs (log2Ratio (S/F)≥1, q-value≥0.8).9 genes were up-regulated, 31 genes and all 7 NATs were down-regulated (Table 4, Fig 1). Among the down-regulated genes, *NT5C3L* (Gene ID 101109197) showed the greatest expression difference (log2Ratio(S/F) = –10.59,q value = 0.89); among the up-regulated genes, *CCNA2* (Gene ID 100144758) showed the greatest expression difference (log2Ratio(S/F) = 6.04, q value = 0.83). 38 significant expression genes had NATs, and only 2 had no found antisense transcripts: *CCNA2* (Gene ID: 100144758) and prolactin-inducible protein homolog (Gene ID: LOC101114011).3 significant DEGs (*AQP8*, Gene ID: 101108013;*Bos d2*,Gene ID:101116281, and *SPRR*,Gene ID: 443313) had significant NATs. All 3 of these genes and their NATs were significantly down-regulated in the super-fine wool group (Table 4).10 DEGs, NATs were used to validate selected differentially expressed transcripts identified from DGE profiling by Real-time PCR. The results from the real-time PCR confirmed the expression pattern of DGEs and NATs at two different groups in Gansu Alpine fine wool sheep.

**Table 4:** Summary of DEGs and NATs between two groups.

Kinds of Transcriptes	GeneID	log2Ratio(S/F)	Probability	Symbol
DEGs	101109197	-10.5912106	0.886583566	NT5C3L
	101116281	-7.40151954	0.969520529	LOC101116281
	101120858	-4.57602606	0.868500902	LOC101120858
	443313	-4.45466762	0.848327046	Small proline-rich
	101109718	-4.16402747	0.835055492	CA4
	101108013	-4.13509115	0.838991854	AQP8
	101118004	-3.99924015	0.86634137	LOC101118004
	101115395	-3.90149163	0.829820677	PGLYRP1
	101114011	-3.8813167	0.855789733	LOC101114011
	101107368	-3.8266707	0.827278443	SLC25A35
	101104026	-3.72227757	0.855311355	S100A8
	101115563	-3.4495271	0.829328632	ABCC11
	101109387	-3.38125687	0.87068777	GLYATL2
	101106121	-3.3527916	0.870715106	LOC101106121
	101115336	-3.29504885	0.839237877	DNASE1L2
	101116409	-3.16407151	0.86568531	LOC101116409
	101113168	-3.1562348	0.826690722	LOC101113168
	100137068	-2.97890634	0.857115521	LOC100137068
	101116799	-2.80442661	0.859794434	LOC101116799
	NM_001009395.1	-2.67922661	0.847588978	-
	101102714	-2.61084761	0.822795364	LOC101102714
	101102540	-2.3882578	0.861489257	RPL39
	101102697	-2.14152458	0.81083593	DNAJC12
	101108654	-2.10679611	0.847930676	LOC101108654
	101116537	-1.89220493	0.803933629	LOC101116537
	101121216	-1.86897593	0.842094473	LOC101121216
	101112716	-1.85095925	0.834194413	KRT7
	101121307	-1.5666126	0.800489312	ACSM3
	101111121	-1.46170294	0.809141108	CD82
	101114256	-1.40678444	0.802238806	ACTG2
	101109430	-1.32738037	0.80115904	KRT1
	101105583	1.377957974	0.802293478	GSDMA
	101110063	1.491887466	0.816426111	HSPA2
	100135694	1.587840025	0.830968782	RPS27A
	443218	1.679295263	0.826485703	FOS
	101113964	1.819875496	0.838185446	PDCD6IP
	101119862	2.058276167	0.826212345	LIPK
	101120443	2.31282643	0.827415122	LOC101120443
	101105188	4.991770667	0.839825597	DAP
	100144758	6.03994716	0.834235416	CCNA2
NATs	101116281	-9.15903036	0.941608145	Bos d2
	443313	-7.587465008	0.819808271	SPRR
	101108013	-7.807354922	0.844108795	AQP8
	101120353	-5.685396543	0.88975588	major allergen I polypeptide chain 2-like
	101120550	-6.896227669	0.975067811	SMC1A
	101113086	-9.556506055	0.957344034	primary amine oxidase
	101113693	-8.661778098	0.914297923	ABP

**Fig 2:**
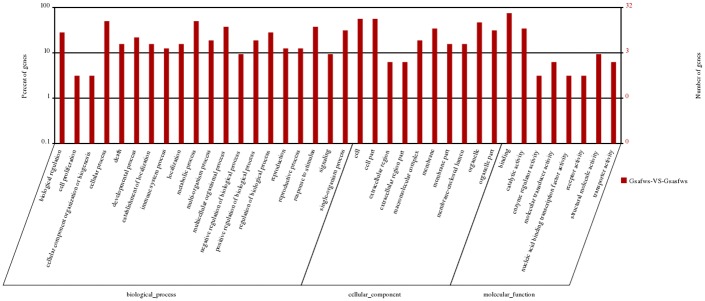
GO functional analysis of DEGs and NATs. The results were summarized in three main categories: biological process, cellular component and molecular function. Among these groups, the terms binding, cell part and metabolic process were dominant in each of the three main categories, respectively.

Gene ontology (GO), Kyoto Encyclopedia of Genes and Genomes (KEGG) and other databases were used for the functional analysis and signaling pathway annotations of these DGEs and NATs. Among all of the significant DEGs and NATs, 44 genes were annotated, and 3 genes located in the genome could not be annotated effectively. In total of 47 DEGs and NATs were summarized as three main GO categories and 38 subcategories (Fig 2). Among the molecular functions category, the top three were involved in binding and catalytic activity. Regarding cellular components, cell part, cell, organelle, membrane, organelle parts were the dominant groups. Within biological processes category, metabolic process and cellular process were the most dominant group. However, no significant enrichment of GO terms was found (corrected P-value >0.05). The pathways that were significantly enriched with significant DEGs and NATs were mainly the lipoic acid metabolism, bile secretion, salivary secretion and ribosome and phenylalanine metabolism pathways (P < 0.05).

## Discussion

Wool is produced via synthetic processes by HFs, which are embedded in the skin of sheep [45]. There are two types of HFs, named primary HF and secondary HF, which are different in appearance and function. Secondary HFs of the fine wool sheep are the main hair follicle and are critical determinants of mean fiber diameter and other wool characteristics. WFD is highly correlated with the size of DP of HF [17], whose origin can be traced to the dermal condensate, one of the earliest features of the developing HF[19]. The charactering of the molecular controls of HF initiation, morphogenesis, branching and growth can facilitate enhanced selection for new sheep breeds with lower WFD [20]. In order to understand the molecular mechanisms of controlling WFD, Adelson *et al*. (2004) constructed three cDNA libraries from fetal and adult sheep skin, obtained 2,345 noredundant EST sequences and identified 61 ESTs expressed in the adult HF, which constituted a high priority candidate gene subset for further work aimed at identifying genes useful as selection markers or as targets for genetic engineering[19]. Norris *et al*. (2005) found 50 up- and 82 down-regulated genes with increased fetal and inguinal expression relative to adult by compared skin gene expression profiles between fetal day 82, day 105, day 120 and adult HF anagen stage using a combined ovine–bovine skin cDNA microarray[20]. However, comparing to sequencing of expressed sequence tags (ESTs) and cDNA microarray, DGE profiling has many unique advantages [46, 47]. It is highly accurate and has a very low detection limit, giving it a very wide range of applications[48],such as detection of new transcripts [49],functional research of non-coding RNA [50, 51]. In this study, six DGE profiling were conducted to analyze the DEGs and NATs in the skin of Gansu Alpine fine wool sheep with different WFD at the same age, gender, and nutrition level during the anagen. More than 4.7 Mb of clean tags were obtained for each sheep, with more than 0.11 Mb of distinct tags. A total of 47 significant DEGs and NATs were detected, including 9 up-regulated genes, 31 down-regulated genes and 7 down-regulated NATs.

The HF comprises several concentric epithelial structures[29]. Wool fiber is enveloped by two epithelial sheaths, known as the inner root sheath (IRS) and the outer root sheath (ORS). At the distal end of the HF, IRS cells undergo apoptosis and liberate the wool fiber [29, 52]. ORS cells contain proliferating cells derived from stem cells in the bulge that feed into the matrix compartment of the bulb[53]. The matrix is a proliferative zone located at the proximal end of the HF surrounding the DP [52]. During the anagen phase, the DP cells are thought to direct the matrix cells to proliferate and differentiate into the multiple cell types that form the wool fiber and its channel, the IRS [9]. During postnatal life, hair follicles show patterns of cyclic activity with periods of active growth and hair production (anagen), apoptosis-driven involution (catagen), and relative resting (telogen) [53–55]. However, Merino HFs are predominantly in anagen throughout growth, different to many animals such as the mouse, rabbit, and guinea-pig [30]. It is also well known that WFD is significantly associated with sizes of DP and matrix in mammals [4, 6, 10–17],which are markedly influenced by genetic[10, 11, 21], physiological[13],nutrition[22], hormones[12] during the anagen phase of the hair cycle. However, the genes specifically controlling the size of DP and matrix remain elusive. In the present study, the up-regulated genes in the super fine wool group included 5 genes associated with the cell cycle and apoptosis: *GSDMA3* [56], *HSPA2* [57], *RPS27A* [58], *PDCD6IP* [59], *DAP*[60], *CCNA2* [61] and *FOS*[62]; the down-regulated genes included 7 genes associated with promoting follicle cell proliferation and differentiation: *KRT1* [63, 64], *KRT7* [65, 66], *HSD11B1* [67, 68], *S100A8* [41, 69, 70], *NT5C3 L*[71] and *DNAJC12* [72]. These genes may through promoting HF cell apoptosis, inhibiting follicle cell proliferation and differentiation, thereby reduce the WFD.

White adiposities, dermal fibroblasts, smooth muscle and endothelial cells of the vasculature, neurons and resident immune cells also surround the HFs in the skin. Recent studies show that the growth of wool fiber is not only affected by the proliferation and differentiation of follicular cells but is also affected by other cells around the follicle [73, 74]. The layer of intradermal adipocytes expands as the HF enters its anagen stage and then thins during telogen [75, 76]. FATP4^*-/-*^, Dgat1^*-/-*^, or Dgat2^*-/-*^ Early B cell factor 1 (Ebf1^*-/-*^) mice have decreased intradermal adipose tissue due to defects in lipid accumulation in mature adipocytes [77–79]. Interestingly, these mice also display abnormalities in skin structure and function, such as hair loss and epidermal hyperplasia. In the present study, in the super fine wool group, it was also determined that the genes related to fat synthesis (*ABCC11*[80], *GLYATL2* [81], and *ACSM3* [82]) were significantly down-regulated and that *LIPK*, which is involved in lipolysis, was significantly up-regulated [83].

Genes with NATs are very common in animal and plant genomes, accounting for 7% to 9% of transcripts in plants and 5% to 30% of transcripts in animals, except for nematodes [84]. The present study indicated that more than 30% of the genes had NATs, which is consistent with the findings of the above mentioned studies. In the present study, correlation analysis was conducted on the sense and antisense transcripts in the DGEs of skin, and the results showed that the correlation coefficients between the sense and antisense transcripts were between 0.533 and 0.557, indicating that the WFD maybe were regulated by both sense and antisense transcripts. NATs regulate sense transcripts through positive or negative feedback by forming a sense-antisense bidirectional structure [85]. In this study, 3 out of 40 significantly differentially expressed genes had significantly different NATs: *AQP8*, *Bos d2*, and *SPRR* (type II small proline-rich protein). *AQP8* belongs to the aquaporin subfamily of the *AQP* family [86]; it is located mainly in a variety of tissues and on the surfaces of acinar cells in various glands and plays important roles in gland secretion and the transportation of water, urea, ammonia and hydrogen peroxide [87–90]. *Bos d 2* belongs to the lipocalin family of proteins[91]. In the skin sections, *Bos d2* was found in the secretory cells of apocrine sweat glands and the basement membranes of the epithelium and HF[92]. It is assumed that *Bos d2* is a pheromone carrier.[92]. The *SPRR* proteins comprise a subclass of specific cornified envelope precursors encoded by a multigene family clustered within the epidermal differentiation complex region [93]. Two *SPRR1*, seven *SPRR2*, one *SPRR3* and one *SPRR4* genes are located within a 300-kb area of the EDC[94, 95] and are expressed in the epidermis, HFs and capillaries [96, 97]. Several studies have suggested that the *SPRRs* are related to increased epithelial proliferation and to malignant processes[98]. Other than functioning as structural proteins, *SPRRs* also regulate gene expression levels, inhibit cell proliferation and promote differentiation [99]. The present study also found that *AQP8*, *Bos d2*, *SPRR* and their antisense transcripts were significantly down-regulated in the super fine wool group, suggesting that *AQP8*, *Bos d2* and *SPRR* and their antisense transcripts were regulated by positive feedback. However, the mechanism by which *AQP8*, *Bos d2* and *SPRR* regulate the WFD needs to be further studied.

## Materials and Methods

### Sheep skin sampling

**Fig 3:**
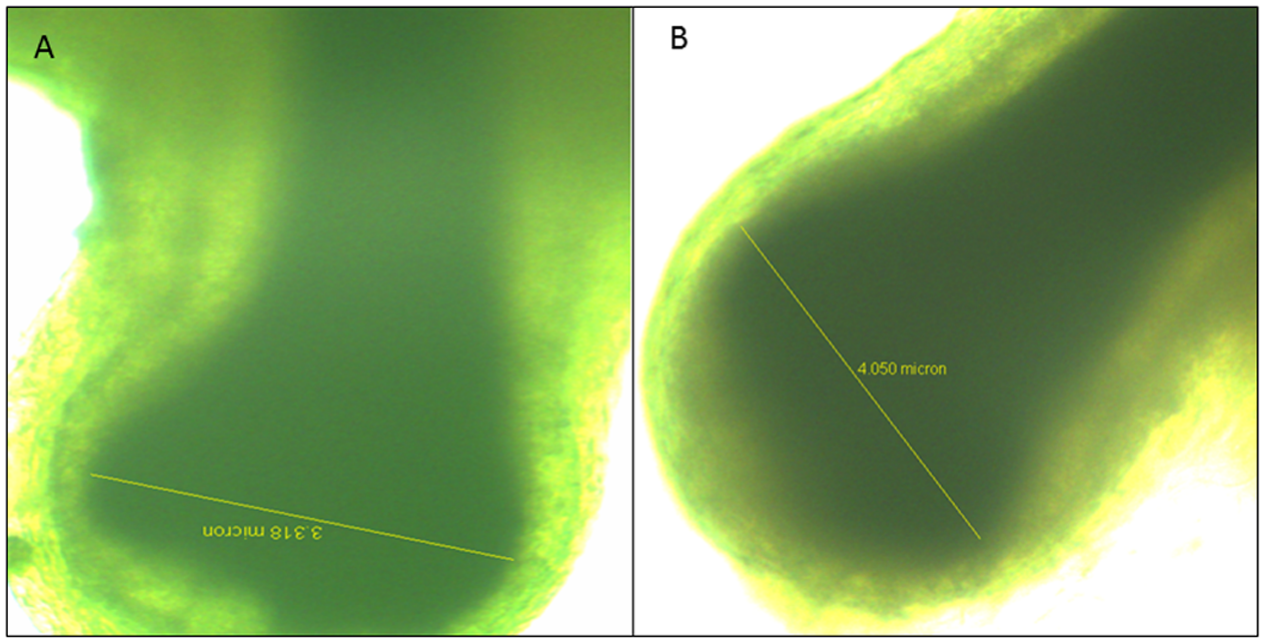
The size of secondary DP cells between two groups. The diameter of super fine wool sheep secondary DP cells = 3.2±0.2μm (A), the diameter of fine wool sheep secondary DP cells = 4.1±0.2μm (B).

Gansu Alpine fine wool sheep were bred in the Huang Cheng District of Gansu Province, China, by cross breeding Mongolian or Tibetan sheep with Xinjiang Fine Wool sheep and then with some fine wool sheep breeds from the Union of Soviet Socialist Republics, such as Caucasian sheep and Salsk sheep. The breed was approved by the Gansu provincial government in 1980. Gansu Alpine fine wool sheep were obtained from a sheep stud farm located in Zhangye city, Gansu Province. All experimental and surgical procedures were approved by the Institutional Animal Care and Use Committee, Lanzhou Institute of Husbandry and Pharmaceutical Sciences, Peoples Republic of China. Six unrelated 3 years old ewes at different WFD, and also as different DP size were selected and divided into super fine wool group (S)(WFD = 18.0±0.5μm; Diameter of secondary DP size = 3.2±0.2μm) and fine wool group (F)(WFD = 23.0±0.5μm; Diameter of secondary DP size = 4.1±0.2μm) (Fig 3). A piece of midside skin (2 mm in diameter) was collected via punch skin biopsy under local anesthesia using 1% procaine hydrochloride immediately placed in liquid nitrogen and stored at -80°C for subsequent analysis.

### Total RNA extraction, library construction and deep sequencing

Total RNA was isolated from the tissues using the RNeasy Maxi Kit (Qiagen, Hilden, GER) according to the manufacturer instructions. RNA quality was verified using a 2100 Bioanalyzer RNA Nanochip (Agilent, Santa Clara, CA, USA), and the RNA Integrity Number (RIN) value was >8.5. Then, the RNA was quantified using the Nano Drop ND-2000 Spectrophotometer (Nano-Drop, Wilmington, DE, USA). Sequence tags were prepared using the Illumina Digital Gene Expression Tag Profiling Kit, according to the manufacturer’s protocol. Sequencing libraries were prepared from 1μg of total RNA using reagents from the NlaIII Digital Gene Expression Tag Profiling kit (Illumina Inc., San Diego, CA, USA). mRNA was captured on magnetic oligo(dT) beads and reverse transcribed into double-stranded cDNA (SuperScript II, Invitrogen, Carlsbad, CA, USA). The cDNA was cleaved using the restriction enzyme *NlaIII*. An adapter sequence containing the recognition sequence for the restriction enzyme *MmeI* was ligated to the *NlaIII* cleavage sites. The adapter-ligated cDNA was digested with *MmeI* to release the cDNA from the magnetic bead, while leaving 17 bp of sequence in the fragment. The fragments were dephosphorylated and purified by phenol–chloroform. A second adapter was ligated at the *MmeI* cleavage sites. Adapter-ligated cDNA fragments were amplified by PCR, and after 15 cycles of linear PCR amplification, 95 bp fragments were purified by 6% TBE PAGE gel electrophoresis, denatured and the single-chain molecules were fixed onto the Illumina Sequencing Chip. A single-molecule cluster sequencing template was created through in situ amplification. Nucleotides labeled with different colors were used to perform sequencing by the sequencing by synthesis method; each tunnel can generate millions of raw reads with a sequencing length of 35 bp.

### Determination of gene expression levels and detection of DEGs and NATs

Sequencing-received raw image data were transformed by base culling into sequence data, which was called raw data. Raw sequences were transformed into clean tags after removed all low quality tags such as short tags (< 21 nt), empty reads, and singletons (tags that occurred only once). Clean tags were classified according to their copy number (as a percentage of the total number of clean tags) and the saturation of the library was analyzed. All clean tags were mapped to sheep reference sequences(version:Oarv3.1) by SOAP2(version:2.21) with default parameters, and allowing one mismatches. To monitor mapping events on both strands, both sense and complementary antisense sequences were included [100].

A preprocessed database of all possible 17 base-long sequences of Oarv3.1 located next to the *NlaIII* restriction site was created as the reference tags, and only one mismatch was allowed. Following the common practice, only clean reads that can be uniquely mapped back to the reference tags were considered ("-r 0" option), and those mapped more than one location were discarded. Remainder clean tags were designed as unambiguous clean tags. When multiple types of remainder clean tags were aligned to the different positions of the same gene, the gene expression levels were represented by the summation of all. The number of unambiguous clean tags for each gene was calculated and then normalized to TPM (number of transcripts per million clean tags) = clean tag number corresponding to each gene number of total clean labels in the sample × 1,000,000 [101, 102].

All TPM of 3 samples each group were integrated, and NOISeq (version: 2.8.0) [103]with default parameters, which is a novel nonparametric approach for the identification of DEGs and NATs, was used to detect the differentially expressed transcripts between super fine wool group (S) and fine wool group (F). To measure expression level changes between two groups, NOISeq takes into consideration two statistics: M(the log_2_-ratio of the two conditions) and D(the absolute value of the difference between conditions). The probability thresholds were *P* ≥ 0.8 and the TMM value in the lower expressed sample was ≥1. The higher the probability, the greater the change in expression between two groups. Using a probability threshold of 0.8 means that the gene is 4 times more likely to be differentially expressed than non-differentially expressed[104].

### Strand-specific real-time quantitative RT-PCR

To confirm the differentially expressed sense and antisense transcripts between super fine wool group and fine wool group, ten genes were randomly selected to verify their expression levels of gene and NATs transcripts in skin by strand-specific qRT-PCR according to the protocol described in Haddad et al. (2007)[105]. Primers for real-time PCR were designed with Primer Express 3.0 (Applied Biosystems) (Table 5). GAPDH was used as a reference control. Real time PCR was performed using SYBR Green master mix (TianGen) on the CFX96 Real-Time System (BioRAD, USA). The reaction was performed using the following conditions: denaturation at 95°C for 3 min, followed by 40 cycles of amplification (95°C for 30s, 60°C for 30s, and 72°C for 30s). Relative expression was calculated using the delta-delta-Ct method.

**Table 5:** Relevant information of gene and primer sequences for strand-specific RT-PCR.

Genes name	Primer sequences (5′→3′)	GenBank accession No	Produce size (bp)
Bos d2	ACAGTAGTGGCACAGAGAGC	XM_004021893.1	136
	TTGTGGTCTTCTCGCCATCA		
SPRR	CAAAAATGCCCTCCTGTGCC	NM_001009773.1	91
	CCTGACCAGATAAAAGCTGATGC		
AQP8	TCATCCTGACGACACTGCTG	XM_004020851.1	146
	ATTCATGCACGCTCCAGACA		
KRT1	CCCTGGATGTGGAGATTGCC	XM_004006320.1	119
	ACTGATGCTGGTGTGACTGG		
KRT7	ACATCAAGCTGGCTCTGGAC	XM_004006333.1	108
	CCACAGAGATGTTCACGGCT		
S100A8	GCTGACGGATCTGGAGAGTG	XM_004002523.1	163
	GAACCAAGTGTCCGCATCCT		
GAPDH	GCTGAGTACGTGGTGGAGTC	NM_001190390.1	136
	GGTTCACGCCCATCACAAAC		
AQP8-NAT	CCTTGGGAAATCCTTGCAGC		137
	GAAAAGCCCGTTGCAGACTC		
Bos d2-NAT	TTGGCGGAAGTATGTCGCAG		112
	CAGCTCAGCCAGAATCGTCA		
SPRR-NAT	CCATGACCCGCTTTGAAGA		132
	CAATGCCAGCAGAAGTGCC		

### GO and KEGG enrichment analysis of differentially expressed transcripts

Gene ontology (GO), an international standardized gene functional classification system, offers a dynamic-updated controlled vocabulary and strictly defined concept to comprehensively describe the properties of genes and their products in any organism[106]. Kyoto Encyclopedia of Genes and Genomes (KEGG) database is a database resource for understanding functions and utilities of the biological system, such as the cell, the organism and the ecosystem from molecular information, especially for large-scale molecular datasets generated by genome sequencing and other high throughput experimental technologies (http://www.genome.jp/kegg/) [107]. all DEGs and NATs were mapped to GO-terms in GO database, looking for significantly enriched GO terms in DEGs comparing to the genome background GOseq R package(version:1.18.0)[108],while significantly enriched metabolic pathways or signal transduction pathways in DEGs and NATs were identified via pathway enrichment analysis using KEGG, public pathway-related database, and comparing with the whole genome background by KOBAS(version: 2.0)[109].

In all tests, P-values were calculated using Benjamini-corrected modified Fisher’s exact test and ≤0.05 was taken as a threshold of significance. The calculating formula is:

P=1-∑i=0m-1MiN-Mn-iNn

Where N is the number of all genes with GO or KEGG annotation; n is the number of DEGs or NATs in N; M is the number of all genes that are annotated to the certain GO terms or specific pathways; m is the number of DEGs or NATs in M.

## Supporting Information

S1 Table Summary of tags mapping to gene & genome and unknown tag. (DOCX)

S2 Table Summary of tags mapping to anti-sense genes and tag-mapped anti-sense genes. (DOCX)
