# Supplementary material for: Exploring Differentially Expressed Genes and Natural Antisense Transcripts in Sheep (Ovis aries) Skin with Different Wool Fiber Diameters by Digital Gene Expression Profiling
Source: PLoS One. 2015 Jun 15;10(6):e0129249. doi: 10.1371/journal.pone.0129249 (PMC4468096; doi:10.1371/journal.pone.0129249)
Supplement: S1 Table — (DOCX) [file pone.0129249.s001.docx]

**S1 Table. Summary of tags Mapping to Gene & Genome and unknown tags.**

| **Sample ID** | **All Tags Mapping to Gene & Genome** | | | | **Unknown Tags** | | | |
| --- | --- | --- | --- | --- | --- | --- | --- | --- |
|  | **Total number** | **Total % of clean tag** | **Distinct Tag number** | **Distinct Tag % of clean tag** | **Total number** | **Total % of clean tag** | **Distinct Tag number** | **Distinct Tag % of clean tag** |
| **5Y127** | 4300237 | 87.45% | 97383 | 81.23% | 617198 | 12.55% | 22503 | 18.77% |
| **5Y212** | 4394852 | 87.32% | 95728 | 81.21% | 638292 | 12.68% | 22152 | 18.79% |
| **5Y339** | 4305181 | 88.63% | 101032 | 83.82% | 552279 | 11.37% | 19493 | 16.17% |
| **65505** | 4386522 | 88.97% | 90904 | 81.98% | 543900 | 11.03% | 19975 | 18.02% |
| **65530** | 4599358 | 88.72% | 97350 | 82.76% | 585017 | 11.28% | 20292 | 17.25% |
| **65540** | 4317964 | 87.38% | 114418 | 79.57% | 623705 | 12.62% | 29382 | 20.43% |
