# Supplementary material for: Exploring Differentially Expressed Genes and Natural Antisense Transcripts in Sheep (Ovis aries) Skin with Different Wool Fiber Diameters by Digital Gene Expression Profiling
Source: PLoS One. 2015 Jun 15;10(6):e0129249. doi: 10.1371/journal.pone.0129249 (PMC4468096; doi:10.1371/journal.pone.0129249)
Supplement: S2 Table — (DOCX) [file pone.0129249.s002.docx]

**S2 Table. Summary of tags mapping to anti-sense gene and tag-mapped anti-sense gene.**

| **Sample ID** | **Unambiguous Tags Mapping to Gene** | | | | **Unambiguous Tag-mapped Genes** | |
| --- | --- | --- | --- | --- | --- | --- |
|  | **Total number** | **Total % of clean tag** | **Distinct Tag number** | **Distinct Tag % of clean tag** | **number** | **% of ref genes** |
| **5Y127** | 286755 | 5.83% | 15957 | 13.31% | 6994 | 36.15% |
| **5Y212** | 302346 | 6.01% | 15693 | 13.31% | 6911 | 35.72% |
| **5Y339** | 287118 | 5.91% | 16226 | 13.46% | 7118 | 36.79% |
| **65505** | 287856 | 5.84% | 14637 | 13.20% | 6730 | 34.79% |
| **65530** | 311060 | 6.00% | 16064 | 13.65% | 6865 | 35.49% |
| **65540** | 306555 | 6.20% | 17167 | 11.94% | 7302 | 37.74% |
